# Eating and Drinking

**Published:** 1854-01

**Authors:** 


### *Eating and Drinking.*

I believe that unwarranted and monstrous errors are propagated, by different writers, on the subject of food and drink. Each man has a whim or hobby, so that it has at length come to the point that if a man will live healthfully to a great age, say a hundred and fifty or two hundred years, he must eat nothing but grapes and drink nothing but rain-water. The gentleman who advocates the grape diet contends that wheat bread ought not to be eaten, that it has too much earth in it, and tends to stiffen a man's joints and muscles half a century sooner than if he subsisted on grapes.

